# Modeling normativity in sustainability: a comparison of the sustainable development goals, the Paris agreement, and the papal encyclical

**DOI:** 10.1007/s11625-017-0504-7

**Published:** 2017-10-29

**Authors:** Gregor Schmieg, Esther Meyer, Isabell Schrickel, Jeremias Herberg, Guido Caniglia, Ulli Vilsmaier, Manfred Laubichler, Erich Hörl, Daniel Lang

**Affiliations:** 1 Faculty of Sustainability, Center for Global Sustainability and Cultural Transformation (CGSC), Leuphana University Lüneburg, Universitätsallee 1, 21335 Lüneburg, Germany; 2 Faculty for Humanities and Social Sciences, Institute of Culture and Aesthetics of Digital Media (ICAM), Center for Global Sustainability and Cultural Transformation (CGSC), Leuphana University Lüneburg, Universitätsallee 1, 21335 Lüneburg, Germany; 3 Faculty of Sustainability, Institute for Ethics and Transdisciplinary Sustainability Research (IETSR), Center for Methods, Center for Global Sustainability and Cultural Transformation (CGSC), Leuphana University Lüneburg, Universitätsallee 1, 21335 Lüneburg, Germany; 4 School of Life Sciences and Center for Biology and Society, Arizona State University, ASU-SFI Center for Biosocial Complex Systems, Tempe, AZ 85287-4501 USA; 5 Faculty for Humanities and Social Sciences, Institute of Culture and Aesthetics of Digital Media (ICAM), Center for Digital Cultures (CDC), Center for Global Sustainability and Cultural Transformation (CGSC), Leuphana University Lüneburg, Universitätsallee 1, 21335 Lüneburg, Germany; 6 Faculty of Sustainability, Institute for Ethics and Transdisciplinary Sustainability Research (IETSR), Leuphana University Lüneburg, Center for Global Sustainability and Cultural Transformation (CGSC), Universitätsallee 1, 21335 Lüneburg, Germany

**Keywords:** Temporal qualities, Dynamical system, Levels, Heterarchy, Norms

## Abstract

The idea of sustainability is intrinsically normative. Thus, understanding the role of normativity in sustainability discourses is crucial for further developing sustainability science. In this article, we analyze three important documents that aim to advance sustainability and explore how they organize norms in relation to sustainability. The three documents are: the Pope’s Encyclical *Laudato Si’*, the *Sustainable Development Goals* and the *Paris Agreement*. We show that understanding the role of different types of norms in the three documents can help understand normative features of both scientific and non-scientific sustainability discourses. We present the diverse system of norms in a model that interrelates three different levels: macro, meso, and micro. Our model highlights how several processes affect the normative orientation of nations and societies at the meso-level in different ways. For instance, individual ethical norms at the micro-level, such as personal responsibility, may help decelerate unsustainable consumerism at the aggregate meso-level. We also show that techno-scientific norms at the macro-level representing global indicators for sustainability may accelerate innovations. We suggest that our model can help better organize normative features of sustainability discourses and, therefore, to contribute to the further development of sustainability science.

## Introduction

 Normativity defines a significant research field within sustainability science, where scientific knowledge and normative orientations are intrinsically linked (Carnau 2011; Miller et al. 2014; Ziegler and Ott 2011). However, it is still unclear how we can understand, or even model, normativity similarly to how we understand and model knowledge about complex biosocial or earth systems (Grunwald 2011, p. 26). In this paper, we suggest ways to understand and model norms in sustainability discourses based on the analysis of three documents: the Pope’s Encyclical *Laudato Si’*, the *Sustainable Development Goals* (SDGs) and the *Paris Agreement* (PA) (see Table 1).

**Table 1:** Synopsis of the three 2015 documents analyzed in this paper

	UN Development Group *Transforming our world: the 2030 Agenda for Sustainable Development (SDGs)* A/RES/70/1 Adopted by the General Assembly: September 25, 2015 Start: January 1, 2016	UN Framework Convention on Climate Change *Paris Agreement (PA)* FCCC/CP/2015/L.9/Rev.1 Sealed: December 12, 2015 Signed: April 22, 2016–April 21, 2017. Start: November 4, 2016	Encyclical Pope Francis *Laudato Si’, on the Care for Our Common Home* Published: June 18, 2015
Authorship	Directed by the United Nations through a deliberative process involving its 193 Member States, as well as global civil society, in order to provide a diversity of perspectives and experience	Drafted during the 21st Conference of the Parties (COP21), November 30, 2015–December 12, 2015 in Paris; France’s foreign minister Laurent Fabius on behalf of the COP21	Pope Francis, drafted by Cardinal Peter Turkson. Precursor summit on April 28, 2015 at the Vatican: “Protect the Earth, Dignify Humanity. The Moral Dimensions of Climate Change and Sustainable Development”—summoned the world religions’ leaders, political leaders, and leading scientists
Words	~ 15.000	~ 16.200	~ 40.500
Languages	Arabic, Chinese, English, French, Russian, Spanish (official languages of the United Nations)	Arabic, Chinese, English, French, Russian, Spanish (official languages of the United Nations)	Arabic, English, French, German, Italian, Polish, Portuguese and Spanish, later Latin and Chinese
Addressee	“[T]his Agenda is a plan of action for people, planet and prosperity; as we embark on this collective journey, we pledge that no one will be left behind” (Preamble); “the future of humanity and of our planet lies in our hands” (§ 53)	Parties of the UNFCCC (member states of the UN)	“[E]very person living on this planet” (p. 4); “enter into dialogue with all people about our common home” (p. 4); “Future generations” (p. 18)
Performance	A resolution is a non-binding intergovernmental agreement “setting out a supremely ambitious and transformational vision” (§ 7). Implementation of: “nationally owned sustainable development strategies”, “enabling international economic environment, including coherent and mutually supporting world trade, monetary and financial systems, and strengthened and enhanced global economic governance”, “availability of appropriate knowledge and technologies globally”, “capacity-building”, “global partnership”.(§ 63)	The Agreement is not legally binding but aims at: “strengthening the global response to the threat of climate change, in the context of sustainable development and efforts to eradicate poverty” (Art. 2.1); “common but differentiated responsibilities and respective capabilities, in the light of different national circumstances” (Art. 2.2); “Facilitative dialogue” (§ 20); “global stocktake” “to assess the collective progress” (Art. 14)	“[A] conversation which includes everyone, since the environmental challenge we are undergoing, and its human roots, concern and affect us all” (p. 14); “act of cooperation with the Creator” (p. 80); “critique of the “myths” of a modernity grounded in a utilitarian mindset (individualism, unlimited progress, competition, consumerism, the unregulated market)” (p. 154); dialogues on “the environment and the international community” (p. 121ff), “new national and local policies” (p. 129ff), “transparency in decision-making” (p. 134ff); “ecological education” (p. 155f)
Time horizons addressed	2016–2030; “seek to build on the Millennium Development Goals” 2000–2015 (Preamble); “decision of great historic significance” (§ 50)	Recalling the UNFCCC in 1992 (Art. 1); first global stocktake in 2023, then every five years (Art. 14); holding the increase in the global average temperature to well below 2 °C above pre-industrial levels (Art. 2); projecting emissions levels for 2030 (§ 17)	“Genesis” (p. 47ff); “the last two hundred years” (p. 39); “future generations” (p. 118ff)
Values	Sustainable development; education; cooperation; capacity-building; universalism; empowerment; the “Goals and targets are integrated and indivisible, global in nature and universally applicable” (§ 55)	“[N]oting the importance for some of the concept of ‘climate justice’ […] of education, training, public awareness, public participation, public access to information and cooperation at all levels” (Annex, p. 21); “environmental integrity”, “transparency”, “accuracy”, “completeness”, “comparability and consistency” (Art. 4)	“Human development” (p. 14), “justice” (p. 10), “universal solidarity” (p. 13), “common good” (p. 40), “scientific consensus” (p. 18), “ecological debt” (p. 36), “differentiated responsibilities” (p. 38), “ecological ethics”, “ecological citizenship” (p. 154)

Works in the field of science studies have shown that social and cultural norms affect scientific theories, institutions, and practices thus challenging simple positivistic conceptions of science (Funtowicz and Ravetz 1993; Gibbons et al. 1994; Putnam 2004; Stengers and Lissack 2004). Norms in the sustainability discourses are both ethical and techno-scientific and relate to relevant actors and entities at different scales—from global and national institutions to local communities and individuals. In our article, we analyze the three important documents produced in 2015 and look at how they structure and organize the ethical and techno-scientific norms that characterize current discourses in sustainability. The norms governing these documents define the broad social, political, and scientific direction of sustainability discourses and interventions in the near future (Nature 2015; Edenhofer et al. 2015).

By focusing our analysis on norms in relation to global development, research programs in sustainability, national policies and individual conduct we carve out a meta-structure of norms. This model-like result conceptualizes the expected performance and impact of the documents in the “age of sustainable development” (Sachs 2015) and helps in the further development of critical understanding of norms in sustainability science.

The broad conception of sustainability and sustainable development embraced here justifies the inclusion of a religious text, such as *Laudato Si’,* in our analysis. We understand *Laudato Si’* as a contribution to the sustainability discourse that goes beyond its own doctrinal and institutional background (Latour 2016). Therefore, we focus heuristically on its ethical rather than religious dimensions (Perkiss and Tweedie 2017).^1^ This emphasis allows us to focus on guiding norms expressed in the Encyclical and related to SD. Our analysis is based on an analytical and yet comprehensive model of norms that integrates the ethics of *Laudato Si’* with the structural importance of normativity in the two UN documents that focus more on techno-scientific issues. Hence, we analyze the role of both ethical and techno-scientific norms in significant contributions to the SD discourse. We show that the three documents are complementary to each other in this perspective. Clarifying normative orientations in sustainability discourses helps to progress towards SD by making more transparent the connection of ethical, socio-political, and scientific dimensions of sustainability (Jerneck et al. 2011; Kläy et al. 2015; Popa et al. 2015). In this sense, our results aim to clarify the potential performance and impact of the three documents in SD discourse. It is still too early to fully assess their actual impact and performance on SD as this implies a retrospective approach.

In what follows, “Material” section gives an overview of the three texts’ genesis and content. “Methods” section outlines our analytical approach for capturing the system of norms embedded within these texts. “Results” section presents the results of the analysis. “Discussion” section discusses our results in relation to specific models used in sustainability science. “Conclusion” section concludes that adequate models in the context of sustainability should incorporate a critical conception of normativity.

## Material

*Laudato Si’, on the Care of Our Common Home* is the second encyclical by Pope Francis. For the first time a Papal encyclical is devoted to environmentalism. Whereas encyclicals are usually addressed to the bishops of the Catholic Church, *Laudato Si’* is addressed to every person on the planet. A summit at the Vatican on April 28, 2015 with the title “Protect the Earth, Dignify Humanity—The Moral Dimensions of Climate Change and Sustainable Development” led to the “Declaration of Religious Leaders, Political Leaders, Business Leaders, Scientists and Development Practitioners”^2^ which foreshadowed the main content of *Laudato Si’*. The Encyclical was then introduced on June 18, 2015 in a press conference at the Vatican attracting extraordinary attention. Speakers were the Ghanaian cardinal of the Roman Catholic Church Peter Turkson, the Eastern Orthodox metropolitan of Pergamon John Zizioulas, who is one of the most influential Orthodox Christian theologians today, the climate scientist and director of the Potsdam Institute for Climate Impact Research (PIK) John Schellnhuber, and Carolyn Woo, CEO and President of Catholic Relief Services and former dean of the Mendoza College of Business, University of Notre Dame, USA.

The report *Transforming our world: the 2030 Agenda for Sustainable Development* is the result of a process that was launched in 2012 at the United Nations Conference on Sustainable Development held in Rio de Janeiro. The member states agreed to develop a set of SDGs that should succeed the UN Millennium Development Goals (MDGs) established in 2000. While the MDGs were mainly geared toward the developing countries, the SDGs apply for all nations. A 30-member Open Working Group (OWG) of the General Assembly was tasked with preparing a proposal for the sustainable development goals. The OWG was established on January 22, 2013. In a new representational mechanism, several countries shared most of the OWG seats. The outcome document of the Rio Conference *The Future We Want* stated that, at the outset, the OWG was to decide on its methods of work, including developing modalities to ensure the involvement of relevant stakeholders and expertise from civil society, the scientific community, and the UN system in its work. The aim was to provide an integrated set of diverse perspectives and experience. On this basis, the intergovernmental negotiations were completed at the UN Sustainable Development Summit in New York (September 25–27, 2015) and the SDGs were adopted by the General Assembly of the United Nations.

The PA was the outcome of the twenty-first session of the Conference of the Parties (COP21, November 30–December 12, 2015) to the United Nations Framework Convention on Climate Change (UNFCCC), an international environmental treaty negotiated in 1992 in order to achieve the “stabilization of greenhouse gas concentrations in the atmosphere at a level that would prevent dangerous anthropogenic interference with the climate system.” The framework convention does not contain any enforcement mechanisms nor does it impose binding limits on greenhouse gas emissions for individual countries. Instead, the framework outlines how specific international treaties (“protocols” or “agreements”) may be negotiated to limit the increase of global average temperature. The PA is such a negotiated outcome. It will enter into force if joined by at least 55 countries representing at least 55 percent of global greenhouse emissions. On 5 October 2016, this threshold was reached.

## Methods

The analytical approach in this paper makes use of the three documents as an entry point for an analysis of the complex system of normativity related to sustainability discourse (Oppermann 2011). To analyze the system of norms in the three documents, we systematically identified the main entities and actors that are guided and influenced by norms. We could identify entities at three different levels: 1. Macro-level, 2. Meso-level, and the 3. Micro-level. We took the three levels as representing the discursive structure of the three texts and focused on specific norms related to the entities on each level as well as their dynamical interactions.

Entities on the macro-level are, for instance, global institutions like the UN, transnational trade organizations, and the Catholic Church while norms are value laden universal ideals such as humanity as a whole and Mother Earth. The ideal of globally valid indicators for measuring SD exemplifies a techno-scientific norm at this level. Entities on the meso-level are nations and societies while relevant norms are accountability, cohesion, or national ownership. Entities on the micro-level are communities, individuals, and more generally subnational entities; an important norm here is moral responsibility.

The three levels of entities and norms are both intra-related (inside one level) and inter-related (between two different levels). We focused largely on inter-level relations and their dynamical properties. Focusing on the relationships between different levels allowed for the emergence of dynamical features related to conceptions of change in the three texts. Thus, entities and respective norms were analyzed with respect to their dynamical effects on other levels within the system. In the course of our analysis, we identified a specific fraction of these relations and the meso-level as the normative core and the focus of action and interventions.

We also focused on dynamical relations that describe processes that refer to acceleration and deceleration vis-a-vis SD. For instance, the acceleration of techno-scientific innovation for climate change mitigation and adaptation is one such process. The deceleration of technical products’ obsolescence rates and private consumerism are examples of a second temporal process. The method of temporal classification of prevalent processes is standard and crucial in complex systems analysis (Simon 2002; Walker et al. 2012). Moreover, studying temporal diversity may lead to operationalizing conceptual models and is important in addressing sustainability problems. This is recognized for example in the fields of sustainable chemistry and health services (Weiser et al. 2017; Sarriot and Kouletio 2015; Cash et al. 2006).

**Fig. 1:**
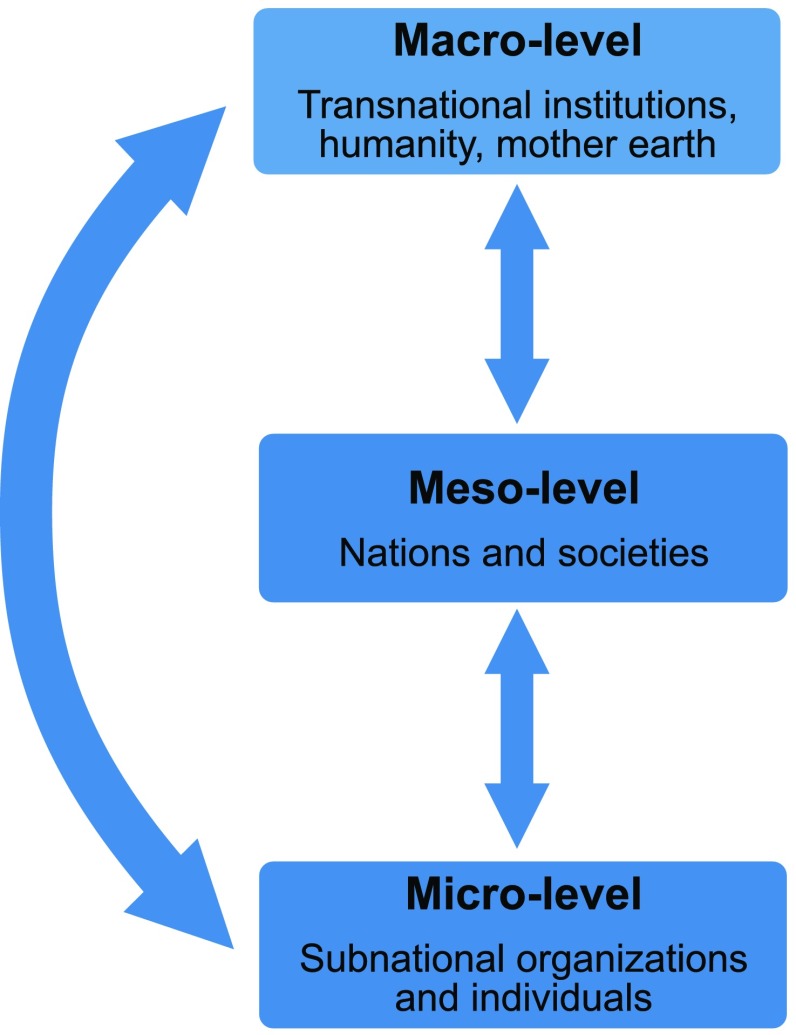
Approach used to capture and model the normative system in the three texts

Figure 1 shows the approach used to capture and model the system of norms in the three texts, focusing on entities and norms at the three different levels as well as the temporal diversity of inter-level processes.

Methodologically, our approach resonates with social-ecological systems (SES) models. These models include socio-political norms and rules as inter-related variables and they can serve as a diagnostic tool for studying sustainability problems (Ostrom 2007). A fundamental feature of such models is their interpretation of the complex systems property of near-decomposability (Ostrom 2007). Near-decomposability means that a system is composed of several subsystems and their dynamic interactions. Furthermore, a system is said to be near-decomposable, if its subsystems are interacting but to a considerable degree autonomously functioning entities (Holland 2012, pp. 15–18; Mitchell 2011, 109–111; Simon 2002, 1962). Figure 1 shows the quasi near-decomposable architecture of our model.

## Results

### The structure of sustainable development

We begin with exposing the structural levels in more detail. By treating entities and norms as descriptive phenomena on each level, we gain a strong perspective on how the three texts conceive of normativity in SD.

#### The macro-level

Universal in scope, the PA states that, “climate change is a common concern of humankind” (PA, preamble). The SDGs address “the human race” as a whole by stating the “critical importance for humanity and the planet” of SD (SDGs, preamble). The Encyclical seeks to “enter into dialogue with all people about our common home” (Enc., 3). All three texts thus relate macro-level entities to universalistic ethical norms and pleas for global frames, such as concern, justice, and commitment. Regarding their scope, the macro-level entities and norms are equally universal and holistic in the three texts. Humanity and the entire planet Earth are the macro-level parameters of normativity in all three documents, while techno-science dominates the PA and the SDGs; metaphysical ethics prevails in the Encyclical. However, the documents differ in the way they interconnect macro-level norms.

In the PA, SD is understood holistically. Pointing out environmental norms at its very core, the PA emphasizes the “importance of ensuring the integrity of all ecosystems” in the sense of safeguarding “Mother Earth” and of achieving “climate justice” (PA, preamble). An intricate connection of ethical and techno-scientific norms occurs when referring to the notions of “ecosystems”, “Mother Earth”, and “justice”. With regard to the techno-scientific side of the PA’s macro-level norms, the focus on numerical restriction of global average temperature rise in Article 2 is even more instructive for understanding the normative architecture. It is evident that it links ethical norms of “justice” and preservation of “ecosystems” with quantifiable information, highlighted through the norm of transparent techno-scientific measurement and the “global stocktake” (PA, 14). The PA presents the correlated scientific process as a means for more equity among the signing parties—as “a facilitative, non-intrusive, non-punitive manner, respectful of national sovereignty, and avoid placing undue burden on Parties” (PA, 13).

The SDGs are in line with the PA, when proposing a “robust, voluntary, effective, participatory, transparent and integrated follow-up and review framework” (SDGs, 72) while stressing the inclusive ethos of leaving no one behind. The envisioned holistic and equitable data system is here called the “global indicator framework” (SDGs, 75).

The Encyclical is guided by super-ordinate norms defined as “categories which transcend the language of mathematics and biology […], intellectual appreciation or economic calculus” (Enc., 11). Summarized under the ethical notion of “love” (Enc., 77) for nature and humankind macro-level norms in the Encyclical countervail the allegedly prevailing “techno-economic paradigm” (Enc., 53, 203), more prominent in the PA and the SDGs.

#### The meso-level

Nations, cultures and societies are the entities at the meso-level. The three texts address techno-scientific and ethical norms at this level in different ways. The Encyclical presents “society” as key entity and “solidarity” as key norm on the meso-level. Whereas in the PA and the SDGs norms strongly relate to techno-scientific issues (refer to “Acceleration and centralization of change” section for the details), the Encyclical promotes “a different cultural paradigm“(Enc., 108). In this formulation climate actions on the meso-level are performed by “society” at large, less so by political entities. The Encyclical even envisions society and culture to be the antipodes of the national state by imposing “regulatory norms” (Enc., 173, 177) on it: “Society […] must put pressure on governments to develop more rigorous regulations, procedures and controls” (Enc., 179).

An altogether different emphasis occurs in the PA and the SDGs. In the PA, “nationally determined contributions” are the most recurrent formula. The related national “climate actions” are meso-level responses to “climate change”. The general focus on the member States that are Parties to the Agreement (PA, preamble) points to the meso-level as the document’s normative focal system. Also the SDGs “will respect national policy“(SDGs, 21). The implementation process outlined by this agenda entails consistency “with the rights and obligations of States under international law“(SDGs, 18) and “national ownership“of the means for SD (SDGs, 46, 66, 74, 76).

#### The micro-level

On the micro-level, the UN documents tend to locate all those entities that represent a non-state approach. Here the PA, for instance, registers subnational entities such as “non-Party stakeholders, including civil society, the private sector, financial institutions, cities and other subnational authorities, local communities and indigenous peoples” (PA, preamble). The SDGs address the same entities on the micro-level although with a stronger emphasis on inclusion compared to the PA. In contrast, the Encyclical explicitly addresses individuals guided by universal norms as the relevant actors at the micro-level.

Altogether, the structural and normative tendencies reveal a key difference in the way the UN documents and the Encyclical refer to structural layers and their interactions that also highlights an overall normative difference: While the UN documents remain elusive on social and explicitly ethical matters and aim for meso-level national institutions, the Encyclical takes a strong ethical stance focusing on the micro-level, especially on individual persons as prime agents of SD.

**Fig. 2:**
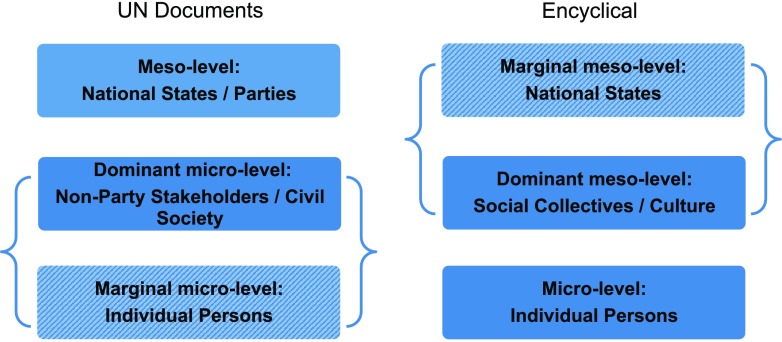
Illustration of discursive dominance and marginalization across the three documents

Figure 2 illustrates the three texts’ differing elaborations of normativity on the micro and meso-level. The brackets in Fig. 2 illustrate the complementarity of the three texts related to the different foci on specific entities. While the UN documents marginalize the role of individuals, they formulate a pronounced regulation of national contributions to SD. Respectively the Encyclical marginalizes national policy matters and introduces a strong account of individual contributions to social concerns in SD.

The argument in Fig. 2 becomes evident in different approaches to education in these three texts. In this regard, the PA defines “climate change education, training, public awareness, public participation and public access to information” (PA, 12) as a politico-scientific norm on the micro-level. The SDGs are complementary here in pointing to the lifestyle aspect of the information norm when pledging that “[b]y 2030, [we] ensure that people everywhere have the relevant information and awareness for sustainable development and lifestyles in harmony with nature” (SDGs, preamble). Lifestyles relate to cultural norms adopted by individuals, but they are not prominent in the SDGs.

This latter aspect is reinforced in the Encyclical. Pope Francis “calls for greater attention to local cultures when studying environmental problems, favoring a dialogue between scientific-technical language and the language of the people. Culture is […] a living, dynamic and participatory present reality, which cannot be excluded as we rethink the relationship between human beings and the environment” (Enc., 143). Thus, micro-level norms rely not only on “scientific information” (Enc., 210) but considerably on “ecological conversion” (Enc., 216-221). The individual “desire to change” does not depend on top-down dissemination of information from the “global stocktake”. Rather micro-level norms in the Encyclical express the individual exigency “to become painfully aware, to dare to turn what is happening to the world into our own personal suffering and thus to discover what each of us can do about it” (Enc.,19). Nevertheless, the Encyclical acknowledges the need for “objective” and “new information” and non-ideological assessment (Enc., 186, 187). But its complementary approach to scientific findings remains a deeply individual and ethical knowledge of individual responsibility. Hence the Encyclical does not lay emphasis on “infinite capacities for activism” (SDGs, 51) based on global information. Instead it highlights relative to the UN norms a provoking and subversive ethics, which “protects human action from becoming empty activism” (Enc., 237).

### The dynamics of change

The next sections analyze temporality in the model by showing that meso-level implementation of SD comes about through dynamics initiated from above (macro-level/top-down) and simultaneously from below (micro-level/bottom-up). The temporal qualities are accordingly top-down acceleration and bottom-up deceleration.

#### Acceleration and centralization of change

The UN documents focus on the implementation of the macro-level techno-scientific ideal of informational transparency on the meso-level. In the PA, implementation is seen as an “accelerating” (PA, 10.5) process. It can “mobilize”, “scale up”, “catalyze” and “increase” meso-level “climate actions”. These processes again depend on “collaborative approaches to research and development, and facilitating access to technology” (PA, 10.5). But the regulative “incentives” deriving from accelerative “mechanisms” refer exclusively to the meso-level national states as parties of the agreement (PA, 5). They do not relate to non-Party stakeholders’ activism on the micro-level. Equally so, the accelerative pattern of implementation applies for the “follow-up and review framework” of the SDGs (SDGs, 36 pp.) depending on a unique universal and “global indicator framework”. Therefore, this macro–meso process of SD can be considered a highly centralistic top-down control mechanism that is assumed to mechanically trickle down and accelerate even collective action on the micro-level.

Notably, this form of control and informational surveillance is translated into an “infrastructure” (SDGs, Goal 9) of technological and financial international facilities operating on “nationally determined contributions”. Accordingly, a just international finance and capacity-building system is presented as a major aim of the PA (PA, §131; see also PA, § 109). Also the norm of transparency relates to a regulative system of “accountability” in political institutions governing the meso-level (SDGs, 16, 17). Mere numerical accounting practices thereby become a normative pivot in the UN documents. As a consequence, accountability as a form of ethical responsibility is reduced to processing and communicating numerical data. Hence, this form of reduction or rationalization corresponds with an accelerated generation of transparent, techno-scientific activity. The latter, according to the UN accounts, is conducive of “adaptive capacity, strengthening resilience and reducing vulnerability” (PA, 7.1) in facing the challenges of SD.

At this point, our analysis reveals an important nuance related to the dynamical quality of acceleration and reduced ethical normativity. The SDGs express this nuance when acknowledging its agenda’s “historic” and “far-reaching” character anno 2015 (SDGs, 2). In the PA and in the Encyclical, the same perspective is formulated as the “long-term global response” (PA, 7.2) related to the macro-level norm of the “long-term common good” (Enc., 178). In this sense, a long-term process corresponds with reducing or slowing down the rate of change at the meso-level, which is challenging to some extent the accelerative reduction of ethical accountability to mere techno-scientific countability. This very significant transformation of accelerative centralistic processes on the meso-level into more decentralized dynamical patterns helps to avoid a lock-in situation in SD. We will show that temporally diverse and truly decentralized meso-level SD depends on autonomous bottom-up processes. As laid out in the following, all three documents indicate that deceleration, the inverse of acceleration, has to be accounted for in relation to the micro-level.

#### Deceleration and decentralization of change

While the SDGs and the PA first and foremost address representative state actors, the Encyclical, when appealing to “every person living on this planet”, follows a much more direct logic. Pope Francis infers that “sustainable and integral development”, and equivalently “authentic social and moral progress” depends on every individual becoming aware of her personal “responsibility” for the ongoing socio-ecological crisis (Enc., 16, 64). The difference relative to the UN accounts is a strong deductive link that establishes the norm of “responsibility” as a direct relation from the universal humanism to the individual person. To state this observation more formally, an autonomous bottom-up micro-level dynamic is introduced by circumventing the meso-level in the first phase of the process rooted in the macro-level. To observe this, one needs to keep in mind the translation of the religious and metaphysical language of the Encyclical into the topology of our model. Otherwise, the dynamics remains vague.

The point is that macro-level normativity is integrated into moral awareness and individual conduct on the micro-level: By “a direct action of God” and by means of the often mentioned “dialogue […] with God himself” (Enc., 81) individuals are said to make “the leap towards the transcendent which gives ecological ethics its deepest meaning” (Enc., 210). The envisioned effect of this normative dynamics is to “develop a different lifestyle and bring about significant changes in society” (Enc., 208), i.e., on the meso-level. The micro-level moral awareness that brings about change on the meso-level correlates with ethical “responsibility” and not with its reduction to techno-scientific “accountability”. The strong emphasis on individuality in this dynamic process implies, however, a decentralized structure of the Encyclical’s normative scheme; every individual is understood as freely responding to a universal (macro-level) normative call.

From a temporal perspective, the individual’s agency is adverse to the top-down acceleration process that is meant to control the “risk” of unforeseeable events through “integrated, holistic and balanced” techno-economic measures (PA, 6.8). In turn, the bottom-up process conveys an inverted normativity that reinforces “social cohesion” as the core of “sustainable and integral development” (Enc., 13). Thus, the Encyclical accounts for SD by means of decelerating the otherwise excessive “acceleration” of human affairs (Enc., 18, 61). The general macro-level norms humanity, divine love, and creation are intended to contribute to a higher sense of collective identity by limiting the pace and speed of individual conduct on the micro-level—an adverse but complementary process relative to the UN documents’ account.

### Summary

**Fig. 3:**
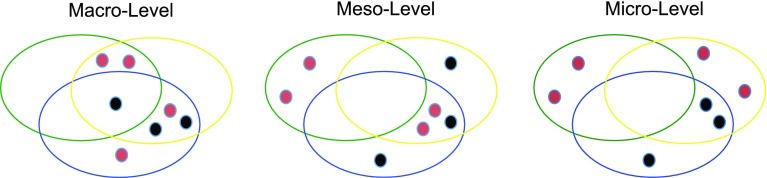
The distribution of the two categories of norms derived from Table 2. Ethical norms (red dots) and techno-scientific norms (black dots) are distributed across the three levels of each document: Encyclica (green), SDGs (yellow), Paris Agreement (blue)

We summarize our results using the following three schemas. Table 2 presents the most important norms in the multi-level perspective. In addition, we index the norms according to the texts they occur in. Figure 3 shows the distribution of the two normative categories in the different texts, i.e., ethical and techno-scientific norms. The figure highlights that there is considerable overlap in the normative orientation of the three texts on the macro-level (see also “The macro-level” section). It also shows that the two UN documents have a rather similar normative approach to meso-level issues, i.e., especially their focus on national sovereignty. Finally, Fig. 3 shows that both SDGs and Encyclical have a much stronger and more balanced normative orientation than the PA on the micro-level. In fact, the SDGs emphasize the importance of “empowerment” and the Encyclical has the topic of “responsibility” as a central one, whereas the Paris Agreement leaves more personal dimensions of change untouched. Therefore, Fig. 3 reveals some normative symmetry between the SDGs and the Encyclical on the micro-level. However, the normative polarization between the Encyclical and the UN documents observed in Fig. 2 dominates the comparison.

**Table 2:** Systematic index of the most significant norms found in the three documents

	Ethical norms	Techno-scientific norms
Macro-level	“Universal”/”global solidarity” (Enz/SDGs) “Climate justice” (PA) “Human rights” (SDGs/PA) “Human dignity” (Enz/SDGs)	Global “indicators” (SDGs)/“stocktake” (PA) “Transparent information” (SDGs/PA) “Global average temperature” (SDGs/PA/Enz)
Meso-level	“Social cohesion” (Enz) “Social and moral progress” (Enz) “Mutual trust” (SDGs/PA) “Common but differentiated responsibilities” (SDGs/PA)	“Accountability” (SDGs) “Capacity-building” (SDGs/PA) “Inform the global stocktake” (PA)
Micro-level	“Responsibility” (Enz) “Empowerment” (SDGs) “Desire to change” (Enz) “Contribution” to change (SDGs)	“Traditional knowledge” (SDGs/PA) “Knowledge of indigenous peoples” (SDGs/PA) “Local knowledge systems” (PA)

The parentheses show the relevant documents

**Fig. 4:**
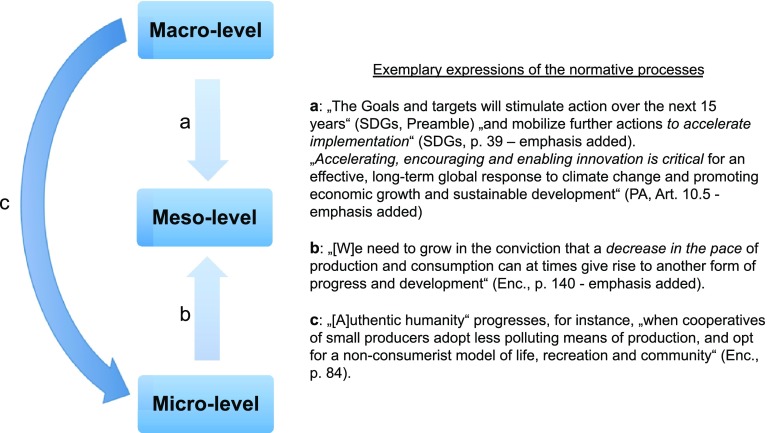
The complex dynamics of change processes in sustainable development according to the three documents’ normative scheme. *a* Stands for techno-scientific acceleration, while *c* and *b* together stand for ethical deceleration of the socio-political realm on the meso-level

Figure 4 shows that the normative dynamics behind change processes at the level of nations and societies (meso-level) are based on a stimulus or incentive aiming at simultaneous acceleration and deceleration. This is a temporal expression of the polarized distribution of ethical and techno-scientific normativity in the three documents. Acceleration of socio-political change is primarily introduced through macro-level techno-scientific and economic innovation policy programs. Deceleration on the other hand is introduced primarily through micro-level action based on individual empowerment, ethical commitment, and responsibility. This result is important because accelerating niche or micro-level activity is often recommended in order to catch up with and eventually counteract or transform fast unsustainable processes on other levels (Ostrom 2009; Geels 2011).

Hence, Fig. 4 shows the dynamics of normativity based on the three texts and their focus on the meso-level. The labeled arrows highlight the most eminent phases or sub-processes of the combined normative system’s dynamics. We call these processes dynamic because they feature different temporal qualities of change across different processes. To differentiate these temporal qualities is crucial to a systemic understanding of sustainability problems and processes (Weiser et al. 2017; Sarriot and Kouletio 2015; Grunwald 2011; Cash et al. 2006). Process a is called techno-scientific acceleration in “Acceleration and centralization of change” section while b and c are called socio-ethical deceleration in “Deceleration and decentralization of change” section. The meso-level is thus the center of a dynamic socio-scientific system including global indicators and accounting practices as well as individual responsible action.

## Discussion

### Topology

As the relationships between macro-level and meso-level are mediated by the micro-level, the normative structure of the sustainability discourse emerging from the three documents is different from the structure of standard and (near-)decomposable (ND) systems. In fact, the latter allows only for direct inter-level relations (Simon 2002). As opposed to this formal hierarchy of ND systems, i.e., box-in-box-in-box systems, the scheme in Fig. 4 possesses main features of a heterarchy type models (McCulloch 1945), also used as models for corporate management in sustainability, such as the Viable System Model (Beer 1959; see also Espinosa and Walker 2011, pp. 8–14). The heterarchy approach to normativity as a cross-scale, cross-level process “can play an important role in engendering shared understanding of different and similar perspectives on how transitions to sustainability may take place” (Peter and Swilling 2014, p. 1616).

### Relation to other models

Figure 4 proposes a heterarchic topology relating fast variables (acceleration processes) to the macro-level and slow variables (deceleration processes) to the micro-level. This result is the exact inverse of prominent model architectures in research on social-ecological or socio-technical systems in the sustainability context (Cash et al. 2006; Ostrom 2007, 2009; Geels 2004; Geels and Schot 2007; Holling 2001; Allen et al. 2014). All these approaches include social phenomena and norms while adopting a fundamental premise related to the biological study of ND systems by Simon (2002, 1962): the relation of fast variables to lower levels, also called niche level, and slow variables to higher levels, also called landscape and sometimes regime level. Here, we propose a complementary approach based on the insight that combining modeling techniques is epistemologically promising in sustainability contexts (Peter and Swilling 2014).

### Transferability of results

The specific heterarchic topology in Fig. 4 depends on the integration of *Laudato Si’* in the analysis. It thus differs from a straightforward SES or socio-technical systems approach. However, Geels (2011) presents a socio-technical model of sustainability transitions that basically also represents a heterarchy. We think that this formal resemblance is necessary in order to transfer our topology of normative dynamics into other contexts of sustainability and improve our understanding of systemic interactions in different sustainability contexts. Therefore, for the purpose of analyzing other sustainability relevant non-scientific documents, especially those related to policy, we suggest that our approach should be directly applicable.

Also, in the international governance context an exciting trend that is increasingly well documented might be studied using our approach. According to this research, there is considerable and growing direct interaction of international environmental bureaucracies (macro-level) with non-state actors (micro-level) for implementing international norms and rules (Hickmann and Elsässer 2017). Specifically, our approach allows us to learn more about the temporal diversity of institutional processes and thus resolve some apparent contradictions within sustainability communities related to techno-scientific and ethical norms. Our approach can also be applied to practical sustainability processes and transformations. It is widely understood that neither these processes nor their outcome can be strictly controlled, “but the speed and focus can be influenced, aiming to facilitate the process” (Espinosa and Walker 2011, p. 276). Knowing how to adapt our knowledge to changing realities means to influence the speed of processes in sustainability contexts. This, however, substantially depends on understanding the normative systems behind related decision-making processes (Geden 2016; Anderies et al. 2013; Jerneck et al. 2011). Confirming this, Sarriot and Kouletio (2015, p. 266) consider “time as a fundamental factor in system adaptation” when it comes to realizing health projects in multi-institutional SD settings.

Finally, our results can contribute to the development of sustainability dialogue design principles. This could help setting up regular and trustful cross-level dialogues about background values, outcome goals, and adequate actions among all project partners and stakeholders each bringing different perceptions of time frames into the dialogue.

Another prominent concept within sustainability that is related to our approach is the concept of leverage points and especially the distinction between deep and shallow leverage points and their respective effects in systems’ transformations. In coupled socio-ecological systems, leverage points often reflect norms and it will be interesting to see if and how the different temporalities identified correspond to such leverage points.

## Conclusion

Socio-political norms are an essential part of current sustainability discourses, both in the form of ethical norms and in the form of techno-scientific norms. Therefore, analysis of the normative structure and dynamics of the three documents’ can help the sustainability community understand how these texts portray and frame the future of sustainability. By modeling normativity in the sustainability discourse, this article will hopefully help better understand how, if actually used to inform policy decisions and practices, the three texts will end up impacting future directions of sustainable development.

Thus, analyzing the roles of norms is highly relevant to sustainability. Making such roles explicit in an adequate model that takes the complexities of multiple different levels and interactions into account is a significant new and challenging research field in sustainability science. In this article, we presented a heterarchic model that deals with norms in the sustainability discourse relying on a comparative analysis of the Pope’s Encyclical *Laudato Si’*, the *Sustainable Development Goals* and the *Paris Agreement.* We argue that, understanding the complexity of normativity in scientific and non-scientific documents dealing with sustainability through our heterarchic model can help the sustainability community deal systematically with normative issues and dimensions in this field.

Because of its analytical resonance with SES and other complexity oriented approaches to sustainability our model can potentially be applied and refined as a new perspective in the field. A vast array of well-documented empirical cases that have so far been analyzed without explicit considerations of the temporal dynamics of norms as variables at different levels can be revisited. Certainly, future research will have to be done on refining this analytical resonance. An advanced and transparent integration of normativity allows for the integration of knowledge and action in order to achieve transformative change in the context of sustainability (Popa 2015; Geden 2016). At the same time, understanding the complexity of normativity generates critical knowledge that can avoid premature “panaceas” (Ostrom 2007).

## Footnotes

1 In fact we could have considered, e.g., the Islamic, the Hindu, and the Buddhist 2015 declarations on climate change as well, because there is a basic interfaith agreement on the normative value of ecology as a global issue and its relation to SD (see http://www.hinduclimatedeclaration2015.org; http://islamicclimatedeclaration.org; https://gbccc.org accessed 28.09.2017).

2 See http://www.endslavery.va/content/endslavery/en/events/protect/declaration.html (Accessed 28 September 2017).

## References

[CR1] Allen CR, Angeler DG, Garmestani AS, Gunderson LH, Holling CS (2014). Panarchy: theory and application. Ecosystems.

[CR2] Anderies JM, Folke C, Walker B, Ostrom E (2013). Aligning key concepts for global change policy: robustness, resilience, and sustainability. Ecol Soc.

[CR3] Beer S (1959). Cybernetics and management.

[CR4] Carnau P (2011). Nachhaltigkeitsethik: Normativer Gestaltungsansatz für eine global zukunftsfähige Entwicklung in Theorie und Praxis.

[CR5] Cash DW, Adger WN, Berkes F, Garden P, Lebel L, Olsson P, Pritchard L, Young O (2006). Scale and cross-scale dynamics: governance and information in a multilevel world. Ecol Soc.

[CR6] Edenhofer O, Flachsland C, Knopf B (2015). Science and religion in dialogue over the global commons. Nat Clim Change.

[CR7] Espinosa A, Walker J (2011). A complexity approach to sustainability: theory and application.

[CR8] Funtowicz S, Ravetz J, von Schomberg R (1993). The emergence of post-normal science. Science, politics and morality.

[CR9] Geden O (2016). The Paris agreement and the inherent inconsistency of climate policymaking. Wiley Interdiscip Rev Clim Change.

[CR10] Geels FW (2004). From sectoral systems of innovation to socio-technical systems: insights about dynamics and change from sociology and institutional theory. Res Policy.

[CR11] Geels FW (2011). The multi-level perspective on sustainability transitions: responses to seven criticisms. Environ Innov Soc Transit.

[CR12] Geels FW, Schot J (2007). Typology of sociotechnical transition pathways. Res Policy.

[CR13] Gibbons M, Limoges C, Nowotny H, Schwartzman S, Scott P, Trow M (1994). The new production of knowledge: the dynamics of science and research in contemporary societies.

[CR14] Grunwald A, Parodi O, Ayestaran I, Banse G (2011). Conflict-resolution in the context of sustainable development. Sustainable development—relationships to culture, knowledge and ethics.

[CR15] Hickmann T, Elsässer J (2017) New alliances in global sustainability governance: international environmental bureaucracies and non-state actors, Paper presented at Interconnections Conference, German Development Institute Bonn, 12–13 May 2017

[CR16] Holland J (2012). Signals and boundaries: building blocks for complex adaptive systems.

[CR17] Holling CS (2001). Understanding the complexity of economic, ecological, and social systems. Ecosystems.

[CR18] Jerneck A, Olsson L, Ness B, Anderberg S, Baier M, Clark E, Hickler T (2011). Structuring sustainability science. Sustain Sci.

[CR19] Kläy A, Zimmermann AB, Schneider F (2015). Rethinking science for sustainable development: reflexive interaction for a paradigm transformation. Futures.

[CR20] Latour B (2016). The immense cry channeled by pope Francis. Environ. Humanit.

[CR21] McCulloch W (1945). A heterarchy of values determined by the topology of nervous nets. Bull Math Biophys.

[CR22] Miller TR, Wiek A, Sarewitz D, Robinson J, Olsson L, Kriebel D, Loorbach D (2014). The future of sustainability science: a solutions-oriented research agenda. Sustain Sci.

[CR23] Mitchell M (2011). Complexity: a guided tour.

[CR24] Nature (2015). Editorial: hope from the Pope. Nature.

[CR25] Oppermann E (2011). The discourse of adaptation to climate change and the UK climate impacts programme: describing the problematization of adaptation. Clim Dev.

[CR26] Ostrom E (2007). A diagnostic approach for going beyond panaceas. Proc Natl Acad Sci.

[CR27] Ostrom E (2009). A general framework for analyzing sustainability of social-ecological systems. Science.

[CR28] Paris Agreement (PA). United Nations Office: Geneva FCCC/CP/2015/L.9/Rev.1. http://unfccc.int/paris_agreement/items/9485.php. Accessed 12 Nov 2015

[CR29] Perkiss S, Tweedie D (2017). Social accounting into action: religion as ‘moral source’. Soc Environ Account J.

[CR30] Peter C, Swilling M (2014). Linking complexity and sustainability theories: implications for modeling sustainability transitions. Sustainability.

[CR31] Popa F, Guillermin M, Dedeurwaerdere T (2015). A pragmatist approach to transdisciplinarity in sustainability research: from complex systems theory to reflexive science. Futures.

[CR32] Pope Francis (2015) Encyclical Letter Laudato Si’ of the Holy Father Francis on Care for Our Common Home. The Holy See: Vatican Press. http://w2.vatican.va/content/dam/francesco/pdf/encyclicals/documents/papa-francesco_20150524_enciclica-laudato-si_en.pdf

[CR33] Putnam H (2004). The collapse of the fact/value dichotomy and other essays.

[CR34] Sachs JD (2015). The age of sustainable development.

[CR35] Sarriot E, Kouletio M (2015). Community health systems as complex adaptive systems: ontology and praxis lessons from an urban health experience with demonstrated sustainability. Syst Pract Action Res.

[CR36] Simon HA (1962). The architecture of complexity. Proc Am Philos Soc.

[CR37] Simon HA (2002). Near decomposability and the speed of evolution. Ind Corp Change.

[CR38] Stengers I, Lissack M (2004). The challenge of complexity: unfolding the ethics of science in memoriam Ilya Prigogine. Emerg Complex Organ.

[CR39] Transforming our world: the 2030 Agenda for Sustainable Development (SDGs), Resolution, A/RES/70/1. http://www.un.org/en/ga/search/view_doc.asp?symbol=A/RES/70/1. Accessed 25 Sept 2015

[CR40] Walker BH, Carpenter SR, Rockstrom J, Crépin A-S, Peterson GD (2012). Drivers, ‘slow’ variables, ‘fast’ variables, shocks, and resilience. Ecol Soc.

[CR42] Weiser A, Lutz LM, Lang DJ, Kümmerer K (2017). Acknowledging temporal diversity in sustainability transformations at the nexus of interconnected systems. J Clean Prod.

[CR41] Ziegler R, Ott K (2011). The quality of sustainability science: a philosophical perspective. Sustain Sci Pract Policy.

